# Role of Cellulose Micro and Nano Crystals in Thin Film and Support Layer of Nanocomposite Membranes for Brackish Water Desalination

**DOI:** 10.3390/membranes9080101

**Published:** 2019-08-15

**Authors:** Mohammed Kadhom, Noor Albayati, Suhaib Salih, Mustafa Al-Furaiji, Mohamed Bayati, Baolin Deng

**Affiliations:** 1 Department of Prostheses, Al-Dour Technical Institute, Northern Technical University, Saladin 41002, Iraq; 2 Department of Science, College of Basic Education, University of Wasit, Azizia, Wasit 52001, Iraq; 3 Department of Chemical Engineering, University of Tikrit, Saladin 41002, Iraq; 4 Environment and Water Directorate, Ministry of Science and Technology, Baghdad 10001, Iraq; 5 Department of Civil and Environmental Engineering, University of Missouri, Columbia, MO 65211, USA; 6 Department of Environmental Engineering, University of Tikrit, Saladin 41002, Iraq

**Keywords:** cellulose nano-crystals, cellulose micro-crystals, reverse osmosis, TFN membranes, desalination

## Abstract

Reverse osmosis is a major process that produces soft water from saline water, and its output represents the majority of the overall desalination plants production. Developing efficient membranes for this process is the aim of many research groups and companies. In this work, we studied the effect of adding cellulose micro crystals (CMCs) and cellulose nano crystals (CNCs) to the support layer and thin film nanocomposite (TFN) membrane on the desalination performance. SEM, TEM, ATR-FTIR, and contact angle measurements were used to characterize the membrane’s properties; and membrane’s performance were evaluated by water flux and NaCl rejection. Filling 2% of CNCs gel in the support layer improved the water flux by +40%, while salt rejection maintained almost the same, around 95%. However, no remarkable improvement was gained by adding CNCs gel to *m*-phenylenediamine (MPD) solution, which was used in TFN membrane preparation. Filling CMCs powder in TFN membrane led to a slight improvement in terms of water flux.

## 1. Introduction

As potable water shortage becomes more severe due to the depletion in traditional resources, the increase in human population, and the climate change, searching for alternative water resources has turned into a priority for scientists and governments [1]. Desalinating saline water is one of the essential solutions for water shortage. Among different approaches for desalination, the reverse osmosis (RO) is the primary process [2]. RO is based on using a selective membrane which allows water molecules to permeate but rejects salts and organics [3]. The state-of-the-art membrane in RO application is the polyamide thin film composite (TFC), which is synthesized by the in situ interfacial polymerization (IP) of MPD and trimesoyl chloride (TMC) on a support layer [4]. Additives such as organic salts [5] and nanoparticles [6] are sometime added into membrane composites to promote their physicochemical properties. To illustrate, different nanosized silica [7], clay [8], zeolite [9], metal–organic frameworks (MOFs) [10] were incorporated to boost the performance membranes in terms of salt rejection and water flux.

Cellulose is a polymer that has been used in a broad range of applications. It exists in enormous quantities in terrestrial and sea plants. Generally, cellulose in its different forms, especially the nanoscale one, is polymeric, water-insoluble, tough, fibrous, light in weight, and is a renewable material, readily available with low cost [11]. Thereby, it could be a good alternative for petroleum derived polymers, notably in bioengineering and bio applications, with a green effect on the environment [12]. The process of shrinking cellulose dimensions down to micro and nano-size went through many steps; however, it was found that fine cellulose has the same structure as the origin. The micro cellulose was involved in many applications, such as in food, pharmaceutical, and paper industries [11]. In recent decades, it was found that cellulose pulps could produce nanofibers when exposing to acid hydrolysis [11] and certain base mixtures [13]. 

In addition to the properties as mentioned above, nanocellulose has high Young’s modulus, high aspect ratio, very good chemical resistance, surface functionalized ability, prime crystallinity, and high surface area [14]. The surface area of the nanocellulose could reach up to 500 m^2^/g via some preparation procedures, while the cellulose pulp surface area ranges from 1 to 4 m^2^/g because amidation, sulfonation, carboxylation, etc., can take place. This dramatic increase is attributed to the increase in hydroxyl groups, which also can be modified with other species [15].

CNCs have been used in many applications, including their uses in water purification as flocculants, absorbents, adsorbents, and membranes [16]. Cellulose was injected in the texture of the mixed matrix membranes (MMMs) for micro- and ultra-filtration, MF and UF, respectively; also, it was filled in the TFN membranes in reverse osmosis and forward osmosis (FO) modes. As a MMMs additive, Bai et al. [17] coated CNCs and cellulose nanofibers (CNFs) on polyethersulfone UF membranes surface where both additives increased the permeability and antifouling properties of the membrane. CNFs grafted membranes showed a slightly better performance than CNCs grafted membranes in terms of water flux, while CNCs grafted membranes showed better performance in terms of reversible and irreversible fouling properties toward humic acid and bovine serum albumin. Daraie et al. [18] incorporated CNCs in 20% polyethersulfone/1% polyvinylpyrrolidone (PVP) MMM, which was used to remove dyes, divalent salt, and mono salt. It was found that by filling 0.8–1.2% CNCs inside the MMM, the water flux value tripled compared with the plain membrane. Additionally, the rejection toward whey protein reached full status, while MgSO_4_ and acid orange 7 rejection increased by 30% and 20%, respectively. It is appropriate to mention that after the fouling test, the water flux recovered 97–99% of its original value. Li et al. [19] modified the polysulfone (PSU) MMM with CNCs and used it for dialysis. It appeared that by filling 0.3 wt.% CNCs in the texture, the water flux doubled; also, lysozyme and urea rejection increased from 42.7% and 62.9% to 70.3% and 90.4%, respectively, while bovine serum albumin rejection maintained almost the same at 96%. Many other studies used the CNCs as additives in the polymeric sheets [20].

TFN membranes incorporated with CNCs in RO were reported very recently. Here, Asempour et al. [21] added different doses of 190 ± 90 nm length and 15 ± 5 nm diameter CNCs to the TMC solution, where their embedding in membranes structure was confirmed by scanning electron microscope (SEM), Atomic force microscopy (AFM), Fourier-transform infrared spectroscopy (FTIR), contact angle and X-ray powder diffraction (XRD) characterizations. Optimally, 0.1% (*w*/*v*) of CNCs increased the water flux from 30 to 63 L/m^2^ h and kept the rejection almost constant at 97.8% under 20 bar pressure. Antifouling property experiments toward 300 ppm bovine serum albumin solution indicated that the 0.1% CNCs membrane decreased the fouling by 11% compared to the virgin membrane. Bai et al. [22] reported that filling 0.02 wt.% CNCs in the polyamide layer increased the water flux by 60%, with maintaining the Na_2_SO_4_ and MgSO_4_ salts rejection around 98.7 and 98.8%, respectively. NaCl rejection increased by increasing CNCs until it reached 22.7% at 0.02% loading. The CNCs incorporated membranes showed higher antifouling and cleaning properties. This improvement was assigned to the increase in hydrophilicity, negative charge, and crosslinking that reduced the pore size. The same group reported a similar study [23] in which they confirmed that CNCs boosted the membranes’ performance when they were used in divalent salt, monovalent salt, and dyes purification. Lastly, Smith et al. [24] filled plain CNCs and 2,2,6,6 tetramethylpiperidine-1-oxyl (TEMPO) oxidized CNCs into the TFC membrane in the RO application. TEMPO-CNCs membranes showed better performance results than CNCs membranes; embedding 0.5 wt.% of TEMPO-CNCs boosted the water flux by 2.6-fold and the salts rejection from 97.5 to 99% compared with the pristine membrane.

In this work, micro- and nano-sized cellulose crystals were incorporated into the inside of the polymeric support layer or TFN membrane. Cellulose is hydrophilic because of the presence of hydroxyl moieties on the structure. The straight long cellulose chain exists often in polymeric bundles due to the van der Waals force and hydrogen bonding among the long molecular chains. Its interactions with polymers, either polysulfone in the supporting layer or polyamide in the thin film, would likely depend on the bundle size of the cellulose fibers. Therefore, it would be interesting to evaluate the impact of CNCs and CMCs on both the supporting polysulfone layer and the polyamide thin film. Findings of filling CNCs into the TFN membrane were compared to the literature, while filling the CMCs into the TFN membrane and embedding CNCs into the support layer texture were first reported in this work. The membrane’s performance was tested using a synthetic brackish water feed and cross flow system. The membrane’s physicochemical properties were examined using the transmission electron microscopy (TEM), SEM, attenuated total reflection Fourier transform infrared (ATR FT-IR) analysis, and contact angle measurement. Different weight ratios of CNCs gel were added while preparing the support layer and thin film membranes, whereas different ratios of CMCs powder were added during the TFN membranes preparation. 

## 2. Experimental Work

### 2.1. Materials 

The CNCs aqueous gel was purchased from Blue Goose Biorefineries Inc. (Saskatoon, SK Canada) that contained 8% CNCs in water, while CMCs powder was purchased from Sigma-Aldrich (St. Louis, MO, USA). MPD (≥99%) and TMC (≥98.5%), the raw reactants for the IP reaction, were purchased from Fisher Scientific (Pittsburgh, PA, USA) and Sigma-Aldrich, respectively. Calcium chloride (CaCl_2_) and isooctane (99%) were obtained from Fisher Scientific, while (1s)-(+)-10-camphorsulfonic acid (CSA, 99%), triethylamine (TEA, ≥99%), and sodium chloride (NaCl, ≥99%) were purchased from Sigma Aldrich. The support layer materials, polysulfone (PSU) of 35,000 molecular weight, 1-Methyl-2-pyrrolidinone (NMP, 99.5%), and N,N-dimethylformamide (DMF, 99.8%), were purchased from Sigma-Aldrich. The water of MPD aqueous solution was provided by a synergy 185 Millipore (Billerica, MA, USA) deionized (DI) water device (18.2 MΩ cm).

### 2.2. Cellulose Micro and Nano Crystal Characterizations

The CNCs were examined by the manufacturing company as listed in Table S1. CMCs were imaged by an FEI Quanta 600 FEG environmental scanning electron microscope (Thermo Fisher Scientific, Hillsboro, OR, USA) device. A powder specimen was spread on an examination adhesive pan and coated with platinum by Emitech (K575x, Kent, UK) sputter coater at 20 milliamps for 1 min; the coating layer thickness was 10 nm.

### 2.3. Preparation of the Support Layer 

The support sheets were prepared by the phase inversion technique. The PSU pullets were dissolved in DMF when the additives (CNCs and CMCs) were incorporated into the TFN membrane. However, PSU pullets were dissolved in NMP when the CNCs gel was added, in different percentages, to the casting solution. DMF was not able to dissolve the CNCs gel, so NMP was used to dissolve the polymer and CNCs to give a homogenous casting solution. In general, the solution was prepared by dissolving 15% PSU in the solvent and heated to 60 °C for 5 h with stirring; the colorless solution was left overnight to cool and degas. A sheet was prepared by sprinkling an aliquot of the mixture on a glass plate by a casting knife (MTI Corp, EQ-Se-KTQ-150) with 130 µm thickness. The plate was then immersed in DI water, the polymer immediately aggregated to form a white sheet which detached from the glass plate in seconds. Ultimately, the prepared sheets were collected, washed free of residue, and stored in DI water at 4 °C until use. 

### 2.4. Preparation of the Thin Membrane

The PSU sheet placed on a glass plate was dried by removing the storage water with a squeegee roller prior to being used as a support layer for the TFN membrane. The support layer was exposed to the MPD aqueous solution for 25 s. MPD aqueous solution consisted of 2% MPD, 1% CSA/TEA organic salt, and 0.01% CaCl_2_, all were in weight ratios. The excess solution was drained off and dried by the squeegee roller. The sheet was left for 2 min in the ambient air to ensure full drying. TMC organic solution, which was prepared by dissolving 0.15% TMC in isooctane, was then poured on top of the PSU sheet that contained MPD roots and left to react for 15 s. The organic solvent residual was removed, and an air knife was utilized to dry the surface. In this procedure, a nanoscale polyamide layer formed by the IP reaction of the two reactants, which served as the active separation layer of the TFC membrane. The fabricated membrane was dried in an oven at 80 °C for 6 min, washed, and stored in DI water at 4 °C before testing.

CNCs gel and CMCs particles were added to the MPD solution due to the hydrophilic nature of the cellulose, which was more compatible with MPD aqueous solution than with the organic phase. The CNCs gel was loaded into the thin membrane in ratios of 0.025, 0.1, 0.125, and 1 wt.%, while the CMCs particles were loaded at 0.1, 0.2, 0.3, 0.6, 0.9, 1.2, and 2 wt.%. Vigorous shaking was applied to ensure the full dispersion of the particles. The CNCs were added to the PSU/NMP solution in ratios of 1, 2, 4, and 6 wt.%.

### 2.5. Characterizations of the Thin Film Membrane

The membrane’s morphology was examined by the SEM using the same procedure used in particles surface examination mentioned in Section 2.1. 

The surface’s functional groups of the membranes were examined by a Nicolet 4700 (Thermo Electron Corporation, Waltham, MA, USA) FT-IR device, where ATR crystal was added to detect the ATR FT-IR spectra. The spectra ranged from 500–4000 cm^−1^, scanned in a rate of 64, and collected at a resolution of 2 cm^−1^. 

The hydrophilicity of the membrane and support layer surfaces was estimated by measuring their contact angle with a DI water drop. A VCA-2500 XE (VCA-2500 XE, AST Products, Billerica, MA, USA) video system from AST products, which measures based on the sessile drop method, was used for this purpose. Each contact angle value reported here is the average of at least ten values, which were estimated at different spots on the specimen surface; the standard deviation of the values represented by the error bars.

Cross-sectional images were captured for the support layer by the transmission electron microscopy (TEM) (JEOL JEM-1400, Peabody, MA, USA) with an accelerating voltage of 120 kV. The specimens were overnight soaked in Eponate 12 resin from Ted Pella Inc. (Redding, CA, USA) and examined by a JEOL JEM-1400 device from JEOL Ltd.

The membranes performance to desalinate a 2000 ppm NaCl brackish solution was evaluated by a cross flow system as illustrated in our previous work [7]. The sample was set in a Millipore Corp stainless steel filtration cell (XX4504700), and a transmembrane pressure of 225 psi was applied for around 6 h at room temperature. The salt rejection was calculated by Equation (1), where its parameters are based on the concentration of the salt in the feed and permeate. A conductivity meter from HACH company (Loveland, CO, USA) was used for this purpose:

(1) $$R=\left(1-\frac{Cp}{Cf}\right)\times 100$$

where *R* is the salt rejection, *Cp* is the permeate conductivity, and *Cf* is the feed conductivity.

The water flux was estimated by Equation (2) and based on the permeate weight accumulation in time:

(2) $$F=\frac{V}{A\times t}$$

where *F* is the water flux (L/m^2^ h), *V* is the desalinated water volume (L), *A* is the membrane’s de facto area (m^2^), and *t* is the desalinated water accumulation time (h). 

## 3. Results and Discussion

### 3.1. Cellulose Micro and Nano Crystal Properties

CNCs gel was obtained from Blue Goose Biorefineries Inc.; it contained 8.0 wt.% CNCs in an aqueous solution and was synthesized by the transition metal catalyzed oxidative process. Table S1 and Figure S1 report the CNCs properties and a TEM image, respectively, as reported in the material data sheet from the manufacturer. The aqueous gel form was used in this study because it is possible that cellulose in gel could be better dispersed in MPD aqueous solution. We aimed to explore if filling CNCs in MPD solution would improve the membrane performance. 

**Figure 1:**
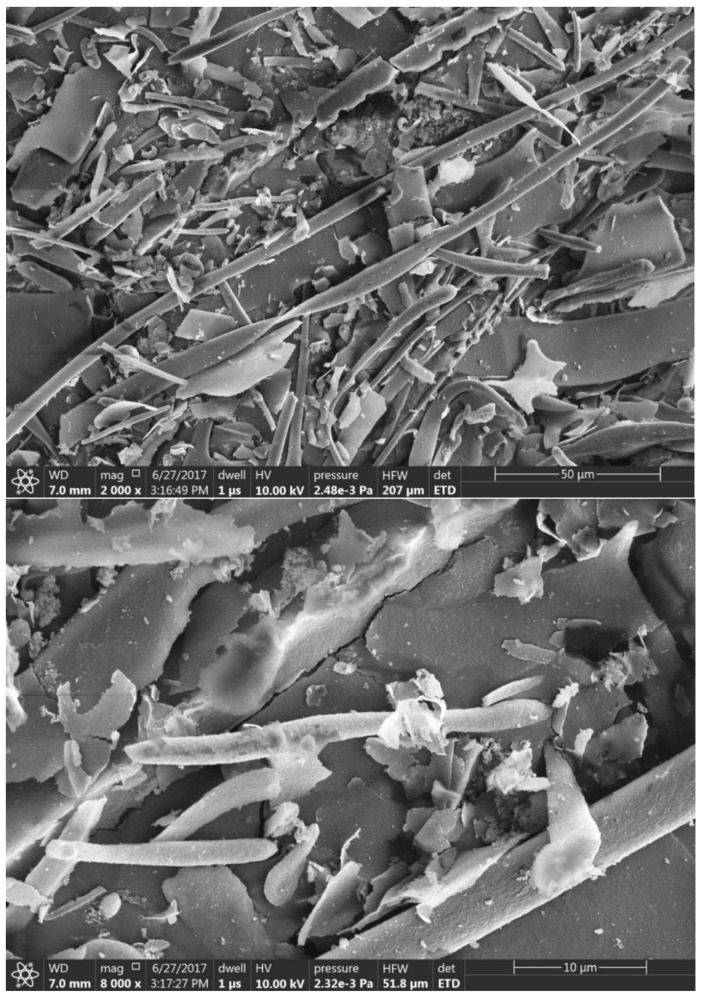
SEM images of CMC powder.

CMCs were obtained from Sigma-Aldrich with an average length of 20 µm. SEM images were collected using the sample preparation method in Section 2.2. and illustrated in Figure 1.

### 3.2. The Thin Film Membranes Properties

**Figure 2:**
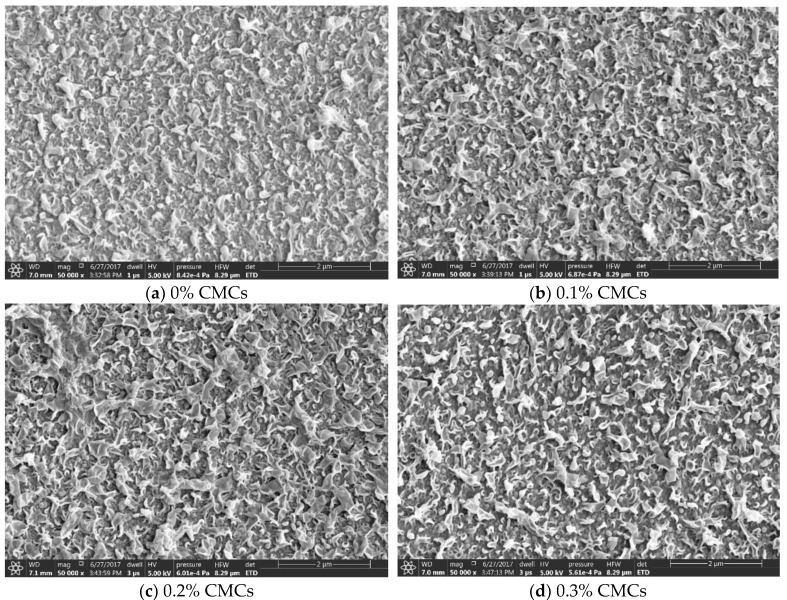

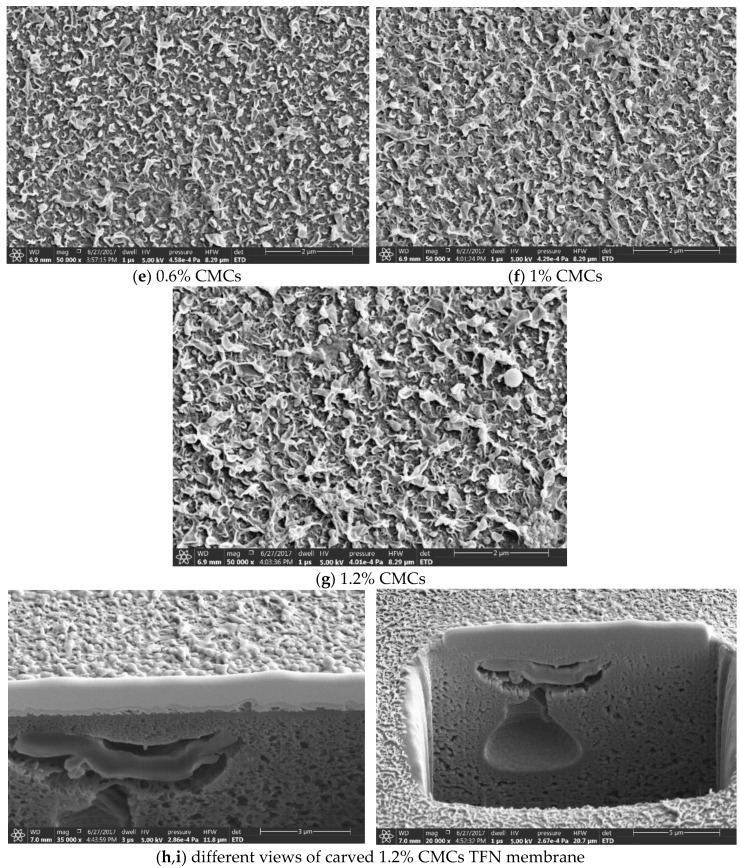
SEM images for TFN membranes filled with different ratios of CMC, (**a**) 0%, (**b**) 0.1%, (**c**) 0.2%, (**d**) 0.3%, (**e**) 0.6%, (**f**) 1%, (**g**) 1.2%, and (**h**,**i**) different views of carved 1.2% CMC membrane.

There were three scenarios for adding cellulose particles into the membranes in this research. The first one was by adding micro size cellulose particles into the thin film nanocomposite membrane. Although the average length of the micro size cellulose was 20 µm, there were some crystals in nano size. Different amounts of CMCs were added to the thin film membrane, 0.1, 0.2, 0.3, 0.6, 1, and 1.2 wt.% of the MPD solution. Figure 2 shows SEM images of the membranes surface with different loading ratios. It is obvious that the TFC membrane (the membrane of 0% loading ratio) had a ridge-and-valley morphology with nodular and leaf-like structure, which is common for this type of membranes. Once the CMCs were loaded, even at low ratios, the surface morphology was noticeably changed, and the protuberances were highly crosslinked. At high loading ratios, e.g., 1.2%, the structure crosslinking was denser due to the increase in the filled crystals. These changes may be attributed to the hydrophilic nature of cellulose or the increase in stress during polyamide formation, which might promote the rate of the IP reaction by enhancing the MPD diffusion into isooctane [8,25]. Another reason could be the swelling phenomenon during membrane’s drying process [26] and the presence of OH groups in cellulose structure that may react with TMC and disturb the reaction conditions [27]. Additionally, the CMCs could increase the miscibility of MPD aqueous solution with TMC organic solution; as a result the MPD could diffuse faster and the membrane would form differently [28]. Finally, the shape of the crystals could contribute to the membrane’s morphological shape [22]. Figure 2i,h show 1.2% CMCs membrane after carving a rectangular piece of the top layer to illustrate the support layer structure following the thin film membrane. The puncture was made by applying a low power electronic beam on the surface for 20 min to avoid surface damage. From images, it appears that the support layer has small and large cavities, in which the water penetrates through. This SL has no cellulose in them and made only from PSU. The effect of cellulose crystals on the support layer will be discussed in detail in a separate section.

**Figure 3:**
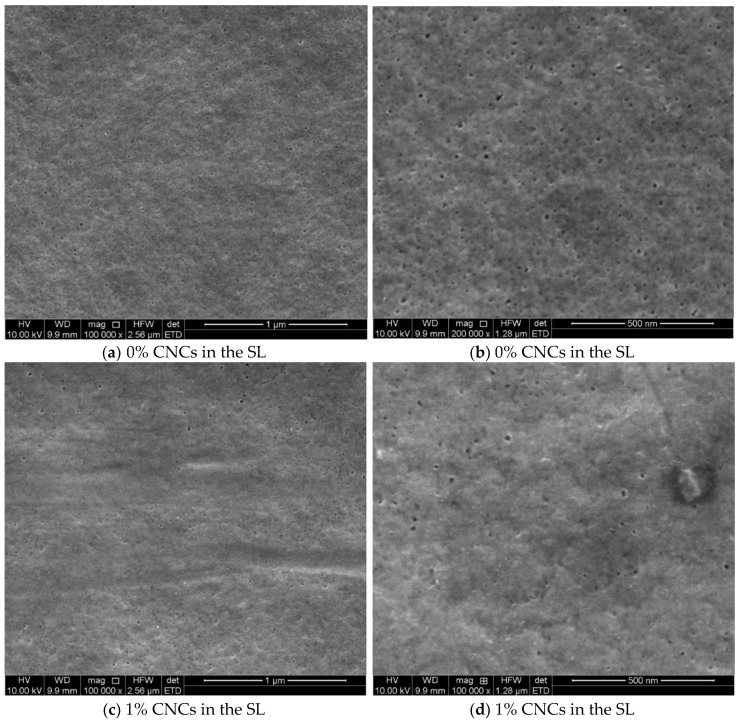

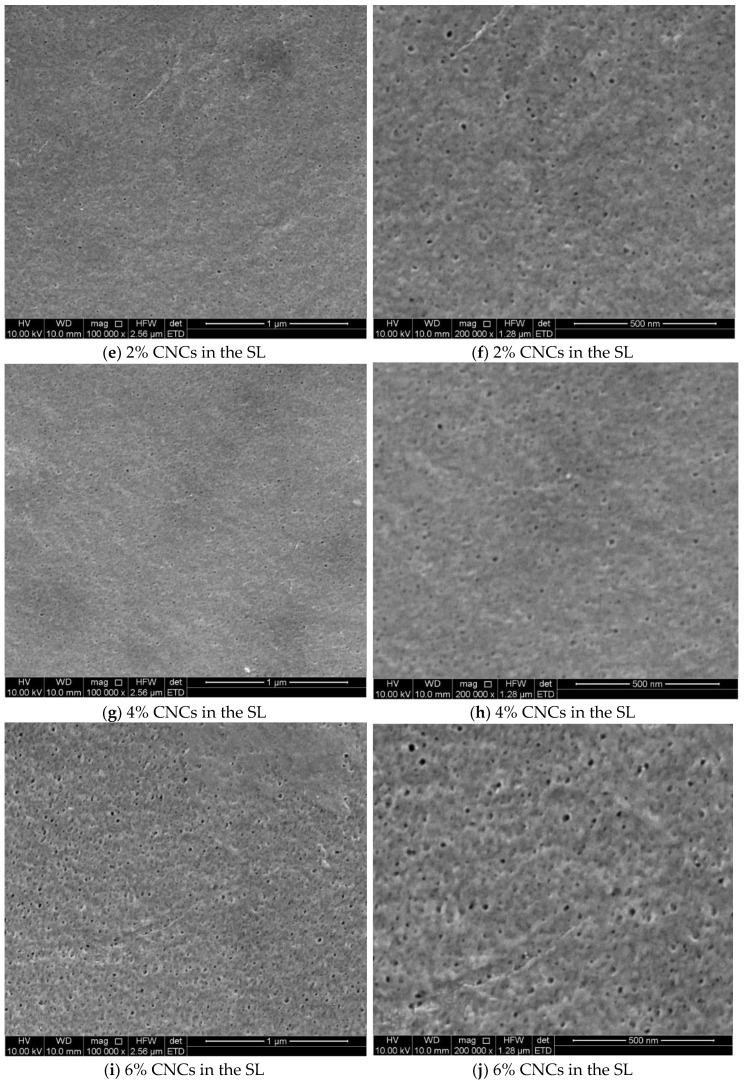
SEM images for the polysulfone support layer filled with different CNCs ratios, (**a**,**b**) 0%, (**c**,**d**) 1%, (**e**,**f**) 2%, (**g**,**h**) 4%, and (**i**,**j**) 6%.

Figure 3 shows images of support layers that were embedded with different loads of cellulose nanocrystals gel (the second scenario). Figure 3a,b were for the top surface pristine support layer, while c and d, e and f, g and h, and i and j were for 1, 2, 4, and 6 wt.%, respectively. It is worth noting that NMP was used as a solvent to disperse the CNCs and dissolve the PSU to prepare the hybrid SLs. In addition to being a powerful solvent, NMP has a high boiling point, low viscosity, low evaporation rate, high thermal and chemical stability, and high water solubility. NMP’s interactions toward unsaturated aromatics and hydrocarbons are high due to the unstable H^+^ in its structure, which made it a protic solvent. NMP is water soluble, which fits its use in dissolving CNCs aqueous gel and makes a homogenous solution. From the figure, it can be recognized that at 0% loading, the pores were not clear in image a; however, at higher resolution, the pores were notable, and the large ones had a diameter of ≈12 nm or less. By mixing 1% CNCs gel with the casting solution, as shown by Figure 3c,d, no large difference was identified. At 2% and 4%, the large pores became wider with a diameter of ≈24 nm or less. Additionally, the number of large pores has increased comparing with lower loading ratios. Finally, at 6% loading, the diameter of the pores increased up to 28.5 nm or less, and the number of large pores also increased. The filled CNCs may increase the hydrophilicity, as the contact angle test will confirm it; thus, the water covered the surface during the phase inversion faster and led to this result. From all images, the pores were well distributed, which is a good indicator to have a uniform TFN membrane after the IP reaction on these support layers, in addition to the possibility of using these polysulfone sheets as membranes in microfiltration and ultrafiltration applications. However, these pores that formed on the top surface are not the effective pores within the membranes, since the membrane’s internal structure is tortuous, and the effective pores are smaller [29]. Additionally, the bottom side pore size is larger than the top side one, as would be confirmed later by TEM images, which agree with previous studies [19,30]. The dimensions were measured by ImageJ software (NIH, Bethesda, MD, USA) and confirmed manually. 

**Figure 4:**
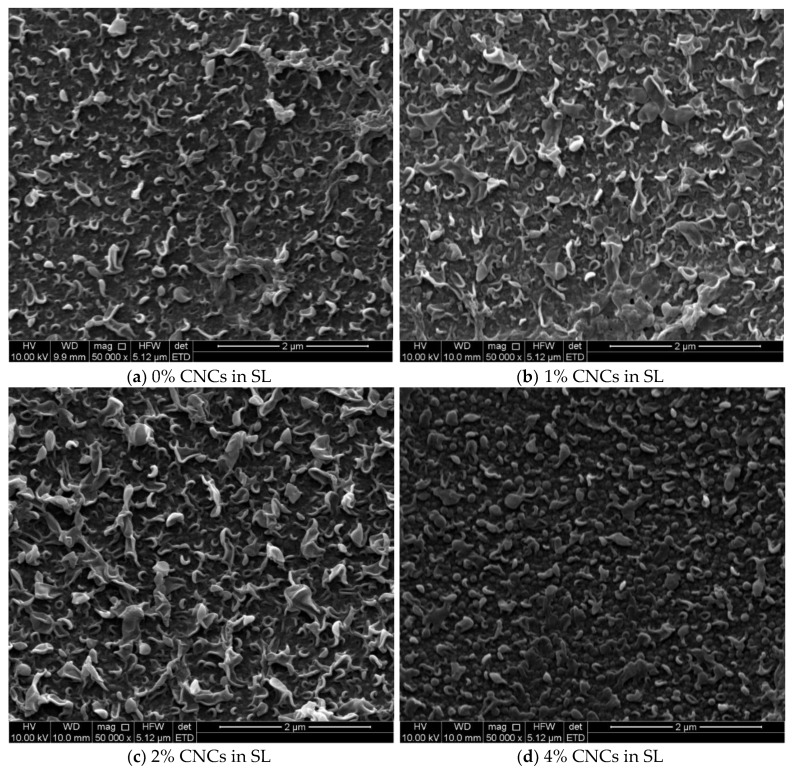

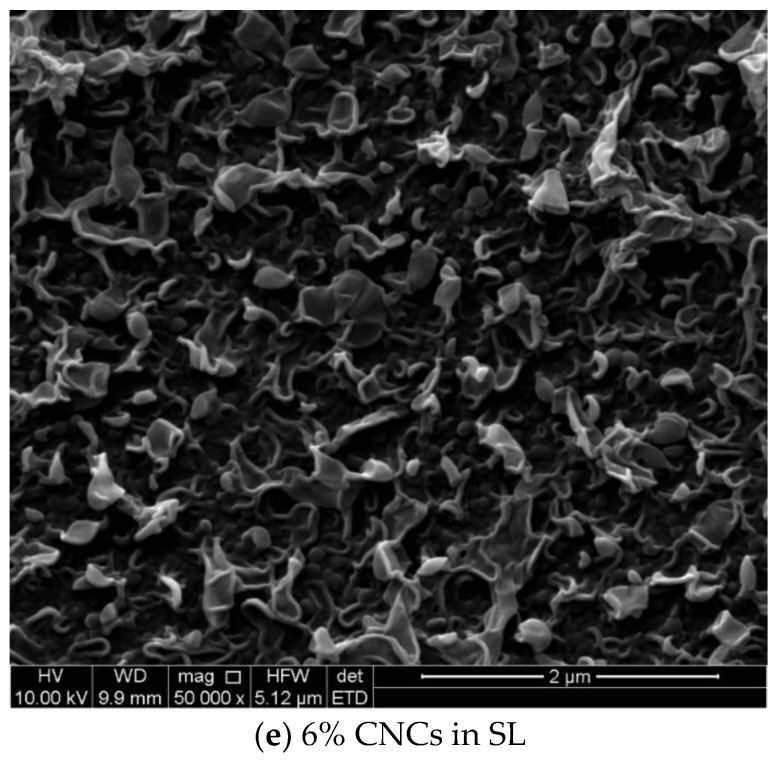
SEM images for the TFN membranes morphology that were synthesized on support layers of different CNCs gel filling ratios, (**a**) 0%, (**b**) 1%, (**c**) 2%, (**d**) 4%, and (**e**) 6%.

Figure 4 shows the TFC membranes that were synthesized on SLs contained different CNCs gel loading in their casting solution. Images a–e of Figure 4 showed the morphology of the TFC membranes made on SLs embedded with 0, 1, 2, 4, and 6% CNCs gel, respectively. Although there were no CNCs filled in the thin film membrane, the CNCs in the support layer influenced the TFC membrane morphology. As the filling ratio augmented, the leaf-like shape polyamide folds started to cross-link with deeper ridge and valley rugose structure. This could be attributed to the OH groups of cellulose on the surface that facilitated the IP reaction and behaved as they were filled into the membrane [22]; the same behavior was observed in Figure 2. 

**Figure 5:**
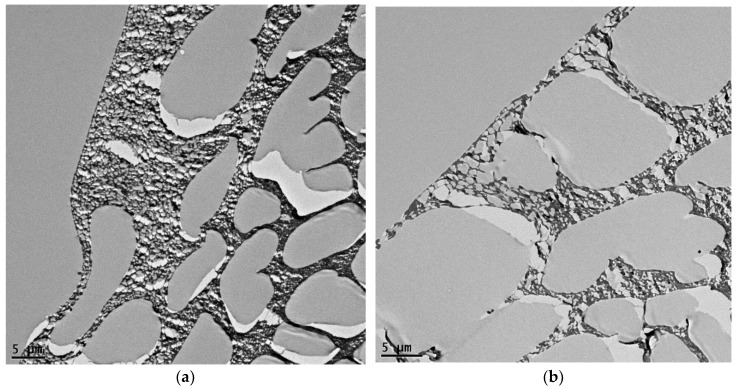

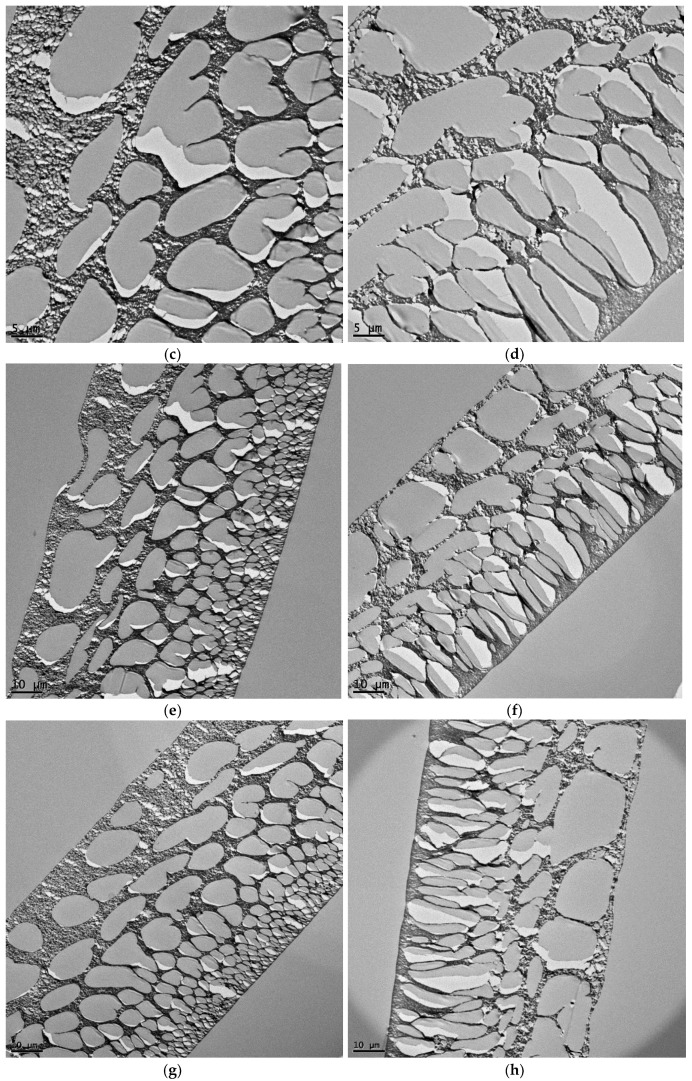
TEM images for the polysulfone support layer (**a**,**c**,**e**,**g**) 0% CNCs; (**b**,**d**,**f**,**h**) 6% CNCs.

Figure 5 shows TEM images in different views for the polysulfone layer before and after adding 6% CNCs gel. The pristine membrane was demonstrated in images a,c,e, and g of Figure 5; while b,d,f, and h of the same figure were for the 6% CNC mixed gel membrane. From the figure, the difference between the two specimens is evident due to the change in the internal structure and the size of cavities. In the pristine sheet, the cavities near the top side were small; however, they became larger and taller with a finger-like shape when the CNCs were added. The bottom side cavities were hollower and irregularly shaped in both types of membranes, this could be imputed to the formation of the top layer, which impedes solvents release and grows up larger macro spacing [19]. The bottom side cavities were more significant when the CNCs were loaded; this could be attributed to the size that CNC’s aqueous gel occupied in the casting solution, which has an affinity to the casting water. When the casting solution immersed in water, NMP and CNC’s gel water vanished within the medium water, although the gel water dispersed faster than NMP. CNCs may also affect the cavities size due to its hydrophilicity, which could accelerate pore formation. These observations are confirmed in previous studies [18,19]. 

**Figure 6:**
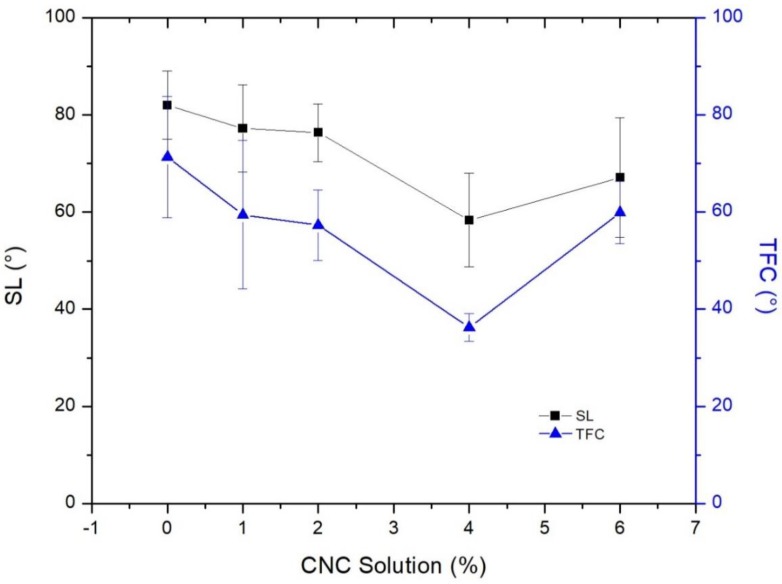
Contact angle for the support layer and thin film composite membrane with different loading ratios of CNCs in the support layer.

Figure 6 shows the contact angle of the SL and TFC membrane before and after adding CNC gel to the casting solution. Although the gel was mixed with the SL casting solution, the thin film membrane hydrophilicity was affected. In general, the SL and TFC membrane hydrophilicity increased by increasing CNCs amount. This could be assigned to the plenty of hydrophilic groups (hydroxyl groups) within the cellulose structure, which increased the surface energy and decreased the contact angle. Increasing the hydrophilicity implies higher water flux and antifouling properties. From figure, the SL contact angle decreased from 82 for the plain sheet to 58.35° when 4% CNCs gel added, while the TFN membrane contact angle decreased from 71.34 for the pristine membrane to 36.26° when 4% CNCs gel added.

Nevertheless, the SL and TFN membrane contact angle increased to 67.12 and 59.93°, respectively, when the CNCs gel addition increased to 6%. This could be attributed to the aggregation of the CNCs at high loading and maldistribution. Here, both SL and TFC membrane had the same behavior, which indicates the influence of SL on the TFC membrane performance. The values of all reported points were the average of ten measured values, whereas the error bars were their standard deviation.

Figure S2 shows the ATR FTIR transmittance spectra for the support layer and TFC membranes with different CNC gel loadings in the support layer casting solution. In both figures, no significant change can be noticed from the reflection of the surface groups even by increasing the CNC gel concentration. This can be attributed to the small amounts of CNCs, the concentration of the crystal in the gel was 8% as we mentioned above. A detailed analysis of the groups was explained in our previous publication [7]. 

Filling CNCs gel in the TFN membrane (the third scenario) was not intensively characterized because it showed low performance results, as shown in the next section.

### 3.3. Membrane’s Performance in Desalination

**Figure 7:**
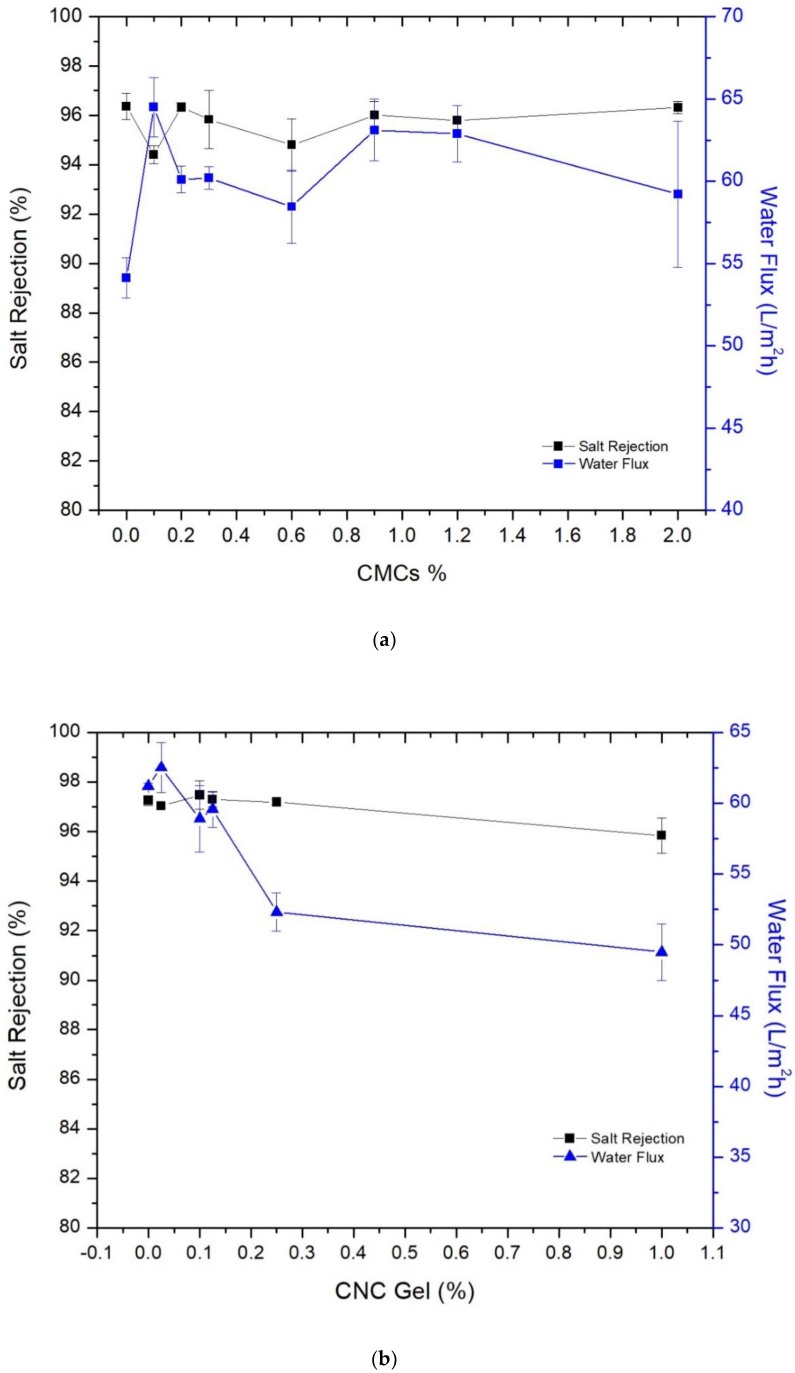

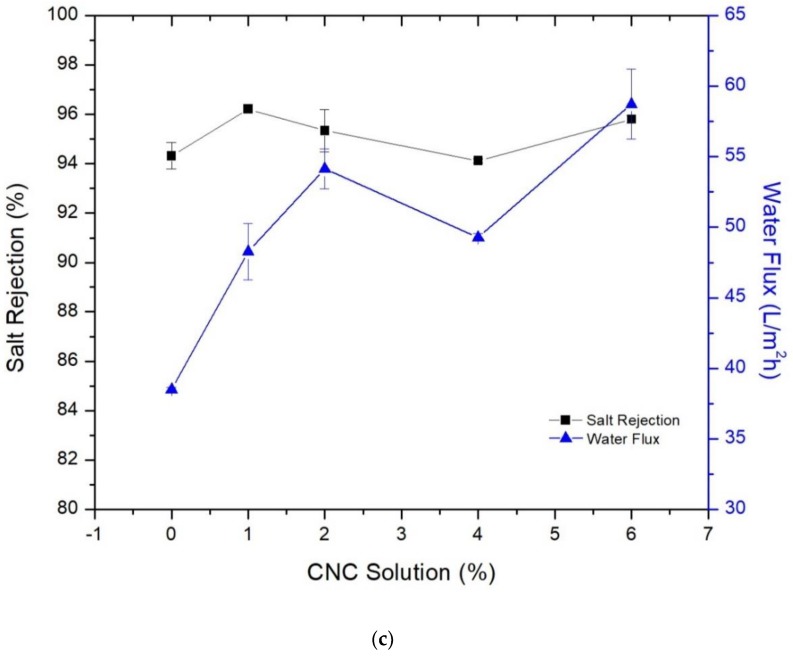
Membranes performance after adding (**a**) CMCs powder to the TFN membrane, (**b**) CNCs gel to the TFN membrane, and (**c**) CNCs gel to the support layer casting solution.

Figure 7 shows the thin film membrane’s performance in terms of salt rejection and water flux toward the NaCl brackish water after filling CMCs and CNCs in the thin film membrane and the support layer. Figure 7a presents the effect of CMCs on the performance; though the average diameter of CMCs is large compared with the desired thickness of the membrane, these particles enhanced the permeate to a certain level without a dramatic sacrificing in rejection. Embedding 0.1% CMCs in the TFC membrane improved the water flux from 54.12 to 64.51 L/m^2^ h, while the rejection decreased from 96.38% to 94.4%, compared with the pristine membrane. This moderate improvement in membrane’s performance could be assigned to the large size of the crystals, which were not fully embedded inside the membrane. However, a further increase in crystals led to unverified behavior, but generally a slight improvement in the performance. This could also be attributed to the crystal size, where increasing the loading ratio did not affect the performance of the membrane.

Figure 7b shows the effect of adding CNCs to the TFN membrane; a modest increase in water flux was recorded by filling 0.025 wt.% of CNCs gel in MPD aqueous solution that used in thin film membrane synthesis. Increasing the filling ratio did not dramatically affect the performance, which is in contrast with the literature [21]. This could be attributed to the use of different sources and types of CNCs. The gel form with lower crystallinity rather than powder was used in this study. Although the CNCs’ gel was in aqueous form and mixed well with MPD-water solution, when it interacted with the TMC-isooctane solution, a non-uniform mix might form due to the different nature of the components, leading to no significant improvement in performance. 

Presumably, the pristine membranes in Figure 7a,b should have the same performance results, but different salt rejections and water fluxes were observed. These differences are attributed to the use of different batches of membrane, where the support layers and thin film membranes were prepared in batches. The preparation conditions could be affected by the ambient temperature, humidity, casting water temperature and level, etc. [31]. Thereby, there are slightly different results for the pristine membranes. From Figure 7a,b, it is observed that filling CMCs in the polyamide membrane had a better impact than filling the CNCs. Incorporating 0.1 wt.% of CMCs led to 19.2% improvement in water flux, with an inconsiderable decrease in NaCl rejection. While 0.025% of CNCs gel (contained 8% of CNCs) increased the water flux by 2.2% with maintaining the salt rejection almost the same. Although the CMCs are larger in size and could not be fully embedded in the membrane comparing to CNCs, they gave better results. Based on our and previous works findings, it can be concluded that cellulose in a form of powder gives better results than the gel when it is filled inside the TFN membrane.

Figure 7c shows the effect of adding CNCs gel with the support layer casting solution on the TFC membrane performance. The effect was evident in this case; adding 2% of CNCs gel to the support layer boosted the water flux of the TFC membrane from 38.5 L/m^2^ h when the plain support layer was used to 54.14 L/m^2^ h, which means a 40% improvement in water flux. Adding 6% to the support layer improved the water flux further to 58 L/m^2^ h and maintained essentially the salt rejection. However, we did not claim it as the best result because it was noticed that further addition of CNC gel led to form tiny holes in the support layer after days of storing in the fridge. This could be due to CNC floc formation, which disrupts the texture of the sheets; as a result, the sheets were not proper for long-term operation. Notwithstanding, the efficiency of the TFC membranes that formed on these SLs did not have a remarkable deterioration as seen from the figure. NaCl rejection fluctuated in a minimal range by increasing the filling ratios, so it was almost stable. There is a concern that these holes may get bigger in long-term operation, then the rejection may drop out.

From the three cases above, it can conclude that the best results were obtained by filling the CNCs gel into the casting solution for support layer preparation. This could be attributed to the strongness of NMP solvent, which dissolved the polymer and CNCs and ended up with a homogenous solution.

## 4. Conclusions

In this research, the impact of cellulose micro- and nano-sized crystals on thin film membrane performance in a desalination application was studied. A commercial CNC gel was used within the TFN membrane and contrasted results with literature were found, which was attributed to the source of the material. CMCs were utilized within the TFN membrane and moderate improvements in terms of performance were obtained, which was likely due to the large size of the crystals that made their filling inside the thin membrane not completed. Finally, the CNCs gel was employed in the support layer texture by using NMP as a solvent; in this case, a remarkable improvement was achieved. Researches are needed to compare the effect of using CNCs that are obtained from different resources, the effect of uptake of MPD and TMC solutions by cellulose [8], and employing CNCs in other desalination aspects [32]. Additionally, using strong solvents, other than NMP, in the casting solution may give different results. Lastly, since NMP can mix with aqueous solutions, studying the addition of water or aqueous solutions of different precursors to NMP-polymer solution could be a good topic of research.

## Supplementary Materials

The following are available online at https://www.mdpi.com/2077-0375/9/8/101/s1.

## Abbreviations

AFM	Atomic force microscopy
ATR FT-IR	Attenuated total reflection Fourier transform infrared
CaCl_2_	Calcium chloride
CMCs	Cellulose micro crystals
CNCs	Cellulose nano crystals
CNFs	Cellulose nanofibers
CSA	(1s)-(+)-10-camphorsulfonic acid
DI	Deionized
DMF	N,N-dimethylformamide
FO	Forward osmosis
FTIR	Fourier-transform infrared spectroscopy
MF	Micro-filtration
MMMs	Mixed matrix membranes
MOFs	Metal–organic frameworks
MPD	*m*-phenyldiamine
NaCl	Sodium chloride
NMP	1-Methyl-2-pyrrolidinone
PSU	Polysulfone
PVP	Polyvinylpyrrolidone
RO	Reverse osmosis
SEM	Scanning electron microscope
SL	Support layer
TEA	Triethylamine
TEM	Transmission electron microscopy
TEMPO	2,2,6,6 Tetramethylpiperidine-1-oxyl
TFC	Thin film composite
TFN	Thin film nanocomposite
TMC	Trimesoyl chloride
UF	Ultra-filtration
XRD	X-ray powder diffraction

## References

[1] Kummu M., Ward P.J., De Moel H., Varis O. (2010). Is physical water scarcity a new phenomenon? Global assessment of water shortage over the last two millennia. Environ. Res. Lett..

[2] Lee K.P., Arnot T.C., Mattia D. (2011). A review of reverse osmosis membrane materials for desalination—Development. J. Membr. Sci..

[3] Kadhom M., Deng B. (2018). Metal-organic frameworks (MOFs) in water filtration membranes for desalination and other applications. Appl. Mater. Today.

[4] Cadotte J. (1981). Interfacially Synthesized Reverse Osmosis Membrane. U.S. Patent.

[5] Zhao L., Ho W.W. (2014). Novel reverse osmosis membranes incorporated with a hydrophilic additive for seawater desalination. J. Membr. Sci..

[6] Lau W., Gray S., Matsuura T., Emadzadeh D., Chen J.P., Ismail A. (2015). A review on polyamide thin film nanocomposite (TFN) membranes: History, applications, challenges and approaches. Water Res..

[7] Kadhom M., Yin J., Deng B. (2016). A Thin Film Nanocomposite Membrane with MCM-41 Silica Nanoparticles for Brackish Water Purification. Membranes.

[8] Kadhom M., Deng B. (2019). Thin film nanocomposite membranes filled with bentonite nanoparticles for brackish water desalination: A novel water uptake concept. Microporous Mesoporous Mater..

[9] Salehi T., Peyravi M., Jahanshahi M., Lau W., Rad A. (2018). Impacts of zeolite nanoparticles on substrate properties of thin film nanocomposite membranes for engineered osmosis. J. Nanoparticle Res..

[10] Kadhom M., Hu W., Deng B. (2017). Thin Film Nanocomposite Membrane Filled with Metal-Organic Frameworks UiO-66 and MIL-125 Nanoparticles for Water Desalination. Membranes.

[11] Habibi Y., Lucia L.A., Rojas O.J. (2010). Cellulose Nanocrystals: Chemistry, Self-Assembly, and Applications. Chem. Rev..

[12] Brinchi L., Cotana F., Fortunati E., Kenny J.M. (2013). Production of nanocrystalline cellulose from lignocellulosic biomass: Technology and applications. Carbohydr. Polym..

[13] Li Y., Zhu H., Xu M., Zhuang Z., Xu M., Dai H. (2014). High Yield Preparation Method of Thermally Stable Cellulose Nanofibers. BioResources.

[14] Abou-Zeid R.E., Khiari R., El-Wakil N., Dufresne A. (2018). Current State and New Trends in the Use of Cellulose Nanomaterials for Wastewater Treatment. Biomacromolecules.

[15] Voisin H., Bergström L., Liu P., Mathew A.P. (2017). Nanocellulose-Based Materials for Water Purification. Nanomaterials.

[16] Mohammed N., Grishkewich N., Tam K.C., Grishkewich N. (2018). Cellulose nanomaterials: promising sustainable nanomaterials for application in water/wastewater treatment processes. Environ. Sci. Nano.

[17] Bai L., Liu Y., Ding A., Ren N., Li G., Liang H. (2019). Surface coating of UF membranes to improve antifouling properties: a comparison study between cellulose nanocrystals (CNCs) and cellulose nanofibrils (CNFs). Chemosphere.

[18] Daraei P., Ghaemi N., Ghari H.S. (2017). An ultra-antifouling polyethersulfone membrane embedded with cellulose nanocrystals for improved dye and salt removal from water. Cellulose.

[19] Li S., Gao Y., Bai H., Zhang L., Qu P., Bai L. (2011). Preperation and characteristics of polysulfone dialysis composite membranes modified with nanocrystalline cellulose. BioResources.

[20] Wang D. (2019). A critical review of cellulose-based nanomaterials for water purification in industrial processes. Cellulose.

[21] Asempour F., Emadzadeh D., Matsuura T., Kruczek B. (2018). Synthesis and characterization of novel Cellulose Nanocrystals-based Thin Film Nanocomposite membranes for reverse osmosis applications. Desalination.

[22] Bai L., Liu Y., Bossa N., Ding A., Ren N., Li G., Liang H., Wiesner M.R. (2018). Incorporation of Cellulose Nanocrystals (CNCs) into the Polyamide Layer of Thin-Film Composite (TFC) Nanofiltration Membranes for Enhanced Separation Performance and Antifouling Properties. Environ. Sci. Technol..

[23] Bai L., Liu Y., Ding A., Ren N., Li G., Liang H. (2019). Fabrication and characterization of thin-film composite (TFC) nanofiltration membranes incorporated with cellulose nanocrystals (CNCs) for enhanced desalination performance and dye removal. Chem. Eng. J..

[24] Smith E.D., Hendren K.D., Haag J.V., Foster E.J., Martin S.M. (2019). Functionalized Cellulose Nanocrystal Nanocomposite Membranes with Controlled Interfacial Transport for Improved Reverse Osmosis Performance. Nanomaterials.

[25] Jeong B.-H., Hoek E.M., Yan Y., Subramani A., Huang X., Hurwitz G., Ghosh A.K., Jawor A. (2007). Interfacial polymerization of thin film nanocomposites: A new concept for reverse osmosis membranes. J. Membr. Sci..

[26] Li Y., Su Y., Dong Y., Zhao X., Jiang Z., Zhang R., Zhao J. (2014). Separation performance of thin-film composite nanofiltration membrane through interfacial polymerization using different amine monomers. Desalination.

[27] Zhang R., Yu S., Shi W., Wang W., Wang X., Zhang Z., Li L., Zhang B., Bao X. (2017). A novel polyesteramide thin film composite nanofiltration membrane prepared by interfacial polymerization of serinol and trimesoyl chloride (TMC) catalyzed by 4-dimethylaminopyridine (DMAP). J. Membr. Sci..

[28] Baroña G.N.B., Lim J., Choi M., Jung B. (2013). Interfacial polymerization of polyamide-aluminosilicate SWNT nanocomposite membranes for reverse osmosis. Desalination.

[29] Kumar S., Nandi B.K., Guria C., Mandal A. (2017). Oil Removal from Produced Water by Ultrafiltration using Polysulfone Membrane. Braz. J. Chem. Eng..

[30] Bai H., Wang X., Zhou Y., Zhangn L. (2012). Preparation and characterization of poly(vinylidenefluoride) composite membranes blended with nano-crystalline cellulose. Prog. Nat. Sci. Mater. Int..

[31] Kadhom M., Deng B. (2019). Synthesis of high-performance thin film composite (TFC) membranes by controlling the preparation conditions: Technical notes. J. Water Process. Eng..

[32] Al-Furaiji M., Benes N., Nijmeijer A., McCutcheon J. (2019). Use of a Forward Osmosis−Membrane Distillation Integrated Process in the Treatment of High-Salinity Oily Wastewater. Ind. Eng. Chem. Res..

