# Supplementary material for: Role of Cellulose Micro and Nano Crystals in Thin Film and Support Layer of Nanocomposite Membranes for Brackish Water Desalination
Source: Membranes (Basel). 2019 Aug 15;9(8):101. doi: 10.3390/membranes9080101 (PMC6723096; doi:10.3390/membranes9080101)
Supplement: Supplementary file 1 [file membranes-09-00101-s001.pdf]

Role of cellulose micro and nano crystals in thin film and support layer of nanocomposite membranes for brackish water desalination

Mohammed Kadhom [1,*], Noor Albayati [2], Suhaib Salih [3], Mustafa Al-Furaiji [4], Mohamed Bayati [5,6] and Baolin Deng [5]

[1] Department of Prostheses, Al-Dour Technical Institute, Northern Technical University, Saladin, Iraq
[2] Department of Science, College of Basic Education, University of Wasit, Azizia, Wasit, Iraq
[3] Department of Chemical Engineering, University of Tikrit, Saladin, Iraq
[4] Environment and Water Directorate, Ministry of Science and Technology, Baghdad, Iraq
[5] Department of Civil and Environmental Engineering, University of Missouri, Columbia, MO 65211, USA
[6] Department of Environmental Engineering, University of Tikrit, Saladin, Iraq
* Correspondence: muhammed.ak@ntu.edu.iq or makbq6@mail.missouri.edu; Tel.: +964-7725-943-915

Table S1. CNCs gel characterizations.

Parameter	*Value*	*Units*	*Test Method*
Solids content	8%	w/w	
Crystallinity index	80%		Segal method
Crystal length	100-150	nm	TEM
Crystal diameter	9-14	nm	TEM
Hydrodynamic diameter	117.2	nm	DLS (dynamic light scattering)
Surface area	≈ 250	m^2/g	
Zeta potential	-30.2	mV	DLS
Carboxyl content	0.129	mmol/g	Conductivity titration + FTIR Image
pH	5.599		
Conductivity	317	µS/cm	

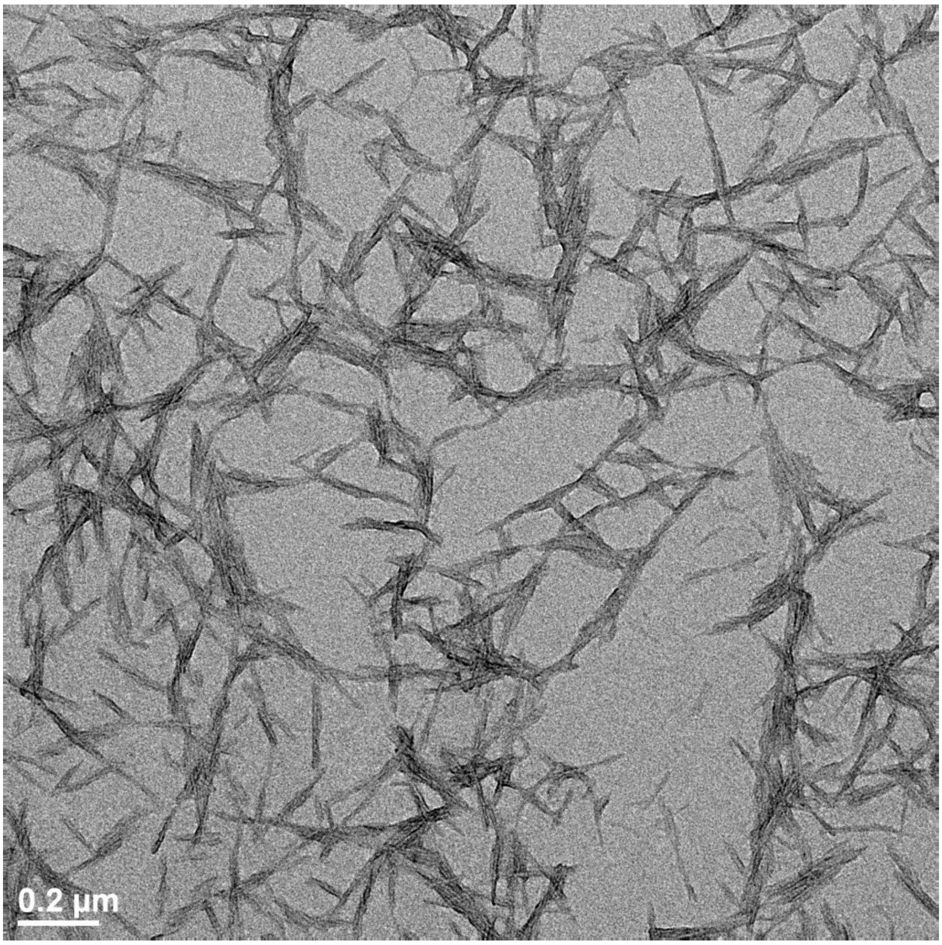

Figure S1. TEM image for CNCs captured by FEI Tecnai 12 BioTwin microscope which was operated at 120 kV. (Blue Goose Biorefineries Inc.).

(a)

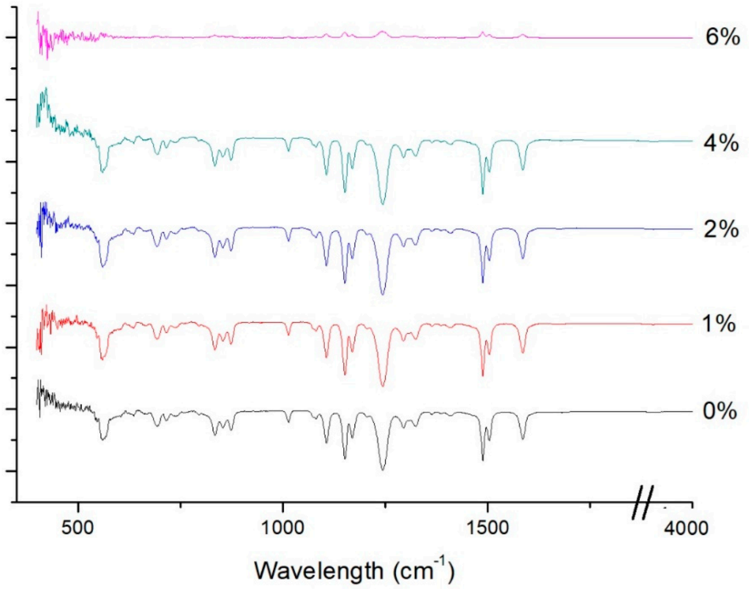

(b)

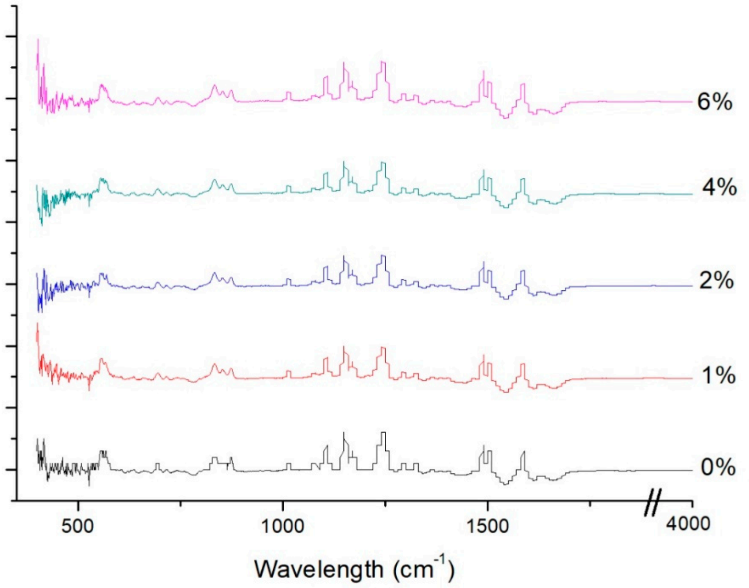

Figure S2. ATR FTIR of (a) SL, (b) TFC membrane with different loading ratios of CNCs in the support layer.
